# Supplementary material for: The Evolution and Characterization of the RNA Interference Pathways in Lophotrochozoa
Source: Genome Biol Evol. 2024 May 7;16(5):evae098. doi: 10.1093/gbe/evae098 (PMC11114477; doi:10.1093/gbe/evae098)
Supplement: evae098_Supplementary_Data [file evae098_supplementary_data.zip › Supplementary_materials.zip!/selected_miRNAs/CrGi_selected_miRNAs/CrGi_NC_047564.1_20988.pdf]

Provisional ID	:	NC_047564.1_20988
Score total	:	252.8
Score for star read(s)	:	3.9
Score for read counts	:	245.4
Score for mfe	:	2.6
Score for randfold	:	1.6
Score for cons. seed	:	-0.6
Total read count	:	493
Mature read count	:	472
Loop read count	:	0
Star read count	:	21

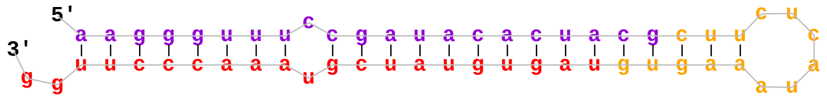

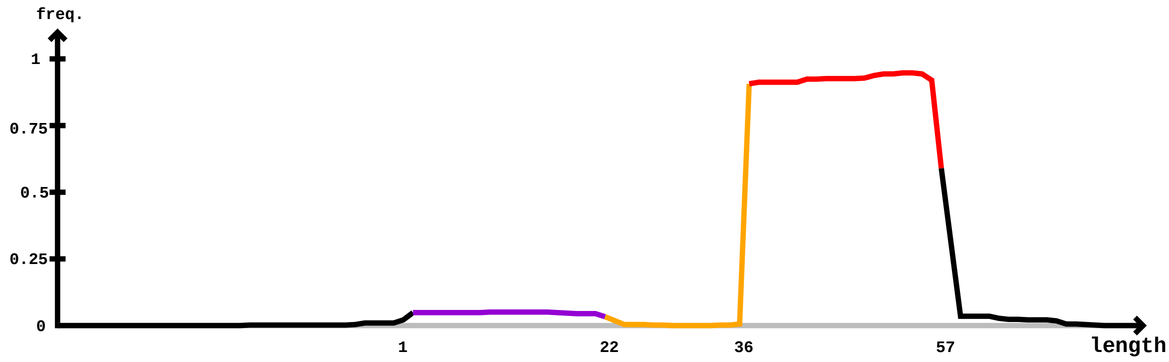

Star Mature

```
5'- aaaagacuuuaggacauuuaccaucuuugcuagccaaggguuuccgauacacuacgcuucucauaaaguguaguguaucguaaacccuuggcuagcgaagaugcacauuua  -3'   obs
    aaaagacuuuaggacauuuaccaucuuugcuagccaaggguuuccgauacacuacgcuucucauaaaguguaguguaucguaaacccuuggcuagcgaagaugcacauuua         exp
    .....................((((((((((((((((((((((.(((((((((((((((......))))))))))))))).))))))))))))))))))))))........    reads     mm        sample
    ..............................uagccaaggguuuccgauaca............................................................    1         0         Ov1
    ..............................uagccaaggguuuccgauacacuacg.......................................................    1         0         Ov1
    ..................................cCaggguuuccgauacacuac........................................................    3         1         Ov1
    ...................................Caggguuuccgauacacuacg.......................................................    2         1         Ov1
    ......................................................................uaguguaucguaaacccuu......................    1         0         Ov1
    ......................................................................Aaguguaucguaaacccuug.....................    1         1         Ov1
    ......................................................................uaguguaucguaaacccuug.....................    20        0         Ov1
    ......................................................................uaguguGucguaaacccuug.....................    1         1         Ov1
    ......................................................................uaguguaucguaGacccuugg....................    1         1         Ov1
    ......................................................................uaguguaucguaaGcccuugg....................    3         1         Ov1
    ......................................................................uaguguaucguaaacccuugg....................    39        0         Ov1
    ......................................................................uaguguaucguGaacccuugg....................    1         1         Ov1
    ......................................................................uaguguaucguaaacccuuggc...................    2         0         Ov1
    ............................................................................aucguaGacccuuggcuagcg..............    1         1         Ov1
    ...................................................................................acccuuggcuagcgaagaug........    1         0         Ov1
    ......................................................................................cuuggcuagcgaagaugAac.....    1         1         Ov1

    ...................................Caggguuuccgauacacuacg.......................................................    8         1         Ov2
    ...........................................ccgauacacuacgcuucuc.................................................    1         0         Ov2
    ......................................................................uaguguaucguaaacccuu......................    1         0         Ov2
    ......................................................................uaguguGucguaaacccuug.....................    1         1         Ov2
    ......................................................................uaguguaucguaaacccuug.....................    17        0         Ov2
    ......................................................................uaguguaucguaGacccuug.....................    1         1         Ov2
    ......................................................................uaguguaucguaaaAccuugg....................    1         1         Ov2
    ......................................................................uaguguaucguaGacccuugg....................    1         1         Ov2
    ......................................................................uaguguaucguaaacccuugg....................    31        0         Ov2
    ......................................................................uaguguaucguaaGcccuugg....................    1         1         Ov2
    ......................................................................uaguguaucguaaacccuuggc...................    2         0         Ov2
    ............................................................................aucguaaacccuuggcuagc...............    2         0         Ov2
    .....................................................................................cccuuggcCagcgaagaug.......    2         1         Ov2
    .....................................................................................Uccuuggcuagcgaagaug.......    1         1         Ov2

    .............................Uuagccaaggguuuccgauac.............................................................    1         1         Mt2
```

Star Mature

```
aaaagacuuuaggacauuuaccaucuuugcuagccaaggguuuccgauacacuacgcuucucauaaaguguaguguaucguaaacccuuggcuagcgaagaugcacauuua
..................................caaggguuuccgauacacuac........................................................ 2 0 Mt2
...................................Caggguuuccgauacacuacg....................................................... 3 1 Mt2
......................................................................uaguguaucguaaacccu....................... 1 0 Mt2
......................................................................uaguguaucguaGacccuu...................... 1 1 Mt2
......................................................................uaguguaucguaaacccuu...................... 4 0 Mt2
......................................................................uaguguaucguaaacccuug..................... 64 0 Mt2
......................................................................uaguguaucguaGacccuug..................... 1 1 Mt2
......................................................................uaguguaucguaaGcccuug..................... 2 1 Mt2
......................................................................uaguguaucguaaacUcuug..................... 1 1 Mt2
......................................................................uaguguaucguaaaUccuug..................... 1 1 Mt2
......................................................................uaguguGucguaaacccuug..................... 2 1 Mt2
......................................................................uaguguaucguGaacccuugg.................... 2 1 Mt2
......................................................................uaguguaucguaaGcccuugg.................... 4 1 Mt2
......................................................................uaguguaucguaaacccuugg.................... 82 0 Mt2
......................................................................uaguguaucguaGacccuugg.................... 1 1 Mt2
......................................................................uaguguGucguaaacccuugg.................... 2 1 Mt2
......................................................................uaguguaucguaaacccuuggc................... 2 0 Mt2
.......................................................................aguguaucguaaacccuug..................... 1 0 Mt2
.......................................................................aguguaucguaaacccuuggc................... 1 0 Mt2
............................................................................aucguaaacccuuggcuagcg.............. 1 0 Mt2
..............................................................................Gguaaacccuuggcuagcgaa............ 1 1 Mt2
..................................................................................aacccuuggcuagcgaagau......... 1 0 Mt2
...................................................................................acccuuggcuagcgaagUu......... 1 1 Mt2
...................................................................................acccuuggcuagcgaagaug........ 1 0 Mt2
...................................................................................acccuuggcuagcgaagaugcG...... 1 1 Mt2
......................................................................................cuuggcuagcgaagaugAaca.... 1 1 Mt2

..................uaccaucuuCgcuagccaaggg....................................................................... 1 1 Mt1
..............................uagccaaggguuuccgauacac........................................................... 1 0 Mt1
..................................cCaggguuuccgauacacuac........................................................ 1 1 Mt1
...................................Caggguuuccgauacacuacg....................................................... 1 1 Mt1
.........................................uuccgauacacuacgcuuc................................................... 1 0 Mt1
...................................................................gCguaguguaucguaaacccuug..................... 1 1 Mt1
.....................................................................guaguguaucguaaacccuug..................... 1 0 Mt1
.....................................................................Uuaguguaucguaaacccuugg.................... 1 1 Mt1
......................................................................uaguguaucguaaacccu....................... 1 0 Mt1
......................................................................uaguguaucguaaacccuu...................... 4 0 Mt1
......................................................................uaguguaucguGaacccuu...................... 1 1 Mt1
......................................................................uaguguaucguGaacccuug..................... 1 1 Mt1
......................................................................uaguguaucguaaacccuug..................... 50 0 Mt1
......................................................................uaguguaucguaaaUccuug..................... 1 1 Mt1
......................................................................uaguguGucguaaacccuug..................... 1 1 Mt1
......................................................................uaguguaucguaGacccuug..................... 1 1 Mt1
......................................................................uaguguaucguaaacccGug..................... 1 1 Mt1
......................................................................uaguguauUguaaacccuugg.................... 1 1 Mt1
......................................................................uaguguaucguGaacccuugg.................... 1 1 Mt1
......................................................................uaguguGucguaaacccuugg.................... 2 1 Mt1
......................................................................uaguguaucguaGacccuugg.................... 3 1 Mt1
......................................................................uaguguaucguaaacccuugg.................... 92 0 Mt1
......................................................................uaguguaucguaaGcccuugg.................... 3 1 Mt1
......................................................................uaCuguaucguaaacccuugg.................... 1 1 Mt1
......................................................................uaguguaucguaaacccuuggc................... 3 0 Mt1
......................................................................uaguguaucguaaGcccuuggc................... 1 1 Mt1
.......................................................................aguguaucguaaacccuuggc................... 1 0 Mt1
............................................................................aucguaaacccuuggcuagc............... 2 0 Mt1
...................................................................................accAuuggcuagcgaagaug........ 1 1 Mt1
```
